# TFCP2 is a transcriptional regulator of heparan sulfate assembly and melanoma cell growth

**DOI:** 10.1016/j.jbc.2023.104713

**Published:** 2023-04-13

**Authors:** Amrita Basu, Rachel N. Champagne, Neil G. Patel, Elijah D. Nicholson, Ryan J. Weiss

**Affiliations:** 1 Complex Carbohydrate Research Center, University of Georgia, Athens, Georgia, USA; 2 Department of Biochemistry and Molecular Biology, University of Georgia, Athens, Georgia, USA

**Keywords:** glycosaminoglycans, heparan sulfate, gene regulation, sulfatase 1, transcription factor, TFCP2, melanoma

## Abstract

Heparan sulfate (HS) is a long, linear polysaccharide that is ubiquitously expressed in all animal cells and plays a key role in many cellular processes, including cell signaling and development. Dysregulation of HS assembly has been implicated in pathophysiological conditions, such as tumorigenesis and rare genetic disorders. HS biosynthesis occurs in a non-template-driven manner in the endoplasmic reticulum and Golgi through the activity of a large group of biosynthetic enzymes. While much is known about its biosynthesis, little is understood about the regulation of HS assembly across diverse tissue types and disease states. To address this gap in knowledge, we recently performed genome-wide CRISPR/Cas9 screens to identify novel regulatory factors of HS biosynthesis. From these screens, we identified the alpha globin transcription factor, TFCP2, as a top hit. To investigate the role of TFCP2 in HS assembly, we targeted TFCP2 expression in human melanoma cells using the CRISPR/Cas9 system. TFCP2 knockout cells exhibited decreased fibroblast growth factor binding to cell surface HS, alterations in HS composition, and slowed cell growth compared to wild-type cells. Additionally, RNA sequencing revealed that TFCP2 regulates the expression of multiple enzymes involved in HS assembly, including the secreted endosulfatase, SULF1. Pharmacological targeting of TFCP2 activity similarly reduced growth factor binding and increased *SULF1* expression, and the knockdown of *SULF1* expression in TFCP2 mutant cells restored melanoma cell growth. Overall, these studies identify TFCP2 as a novel transcriptional regulator of HS and highlight HS–protein interactions as a possible target to slow melanoma growth.

Heparan sulfate proteoglycans (HSPGs) are glycoproteins ubiquitously expressed on the cell surface and in the extracellular matrix of all animal cells. One or more heparan sulfate (HS) chains are covalently attached to these core proteins and are assembled in the endoplasmic reticulum and Golgi apparatus *via* polymerization of disaccharide subunits containing *N*-acetyl-D-glucosamine and D-glucuronic acid (GlcA). These repeating subunits are *N*- and *O*-sulfated at different positions through the activity of a large group of sulfotransferase enzymes (NDSTs, HS2ST1, HS6STs, HS3STs), which endow the chains with immense structural heterogeneity across different mammalian tissues and cell types (1). Highly sulfated HS domains can vary in their size and composition, and additional secreted factors, including the endosulfatases SULF1 and SULF2, can remodel HS structure and function at the cell surface through the removal of key 6-*O*-sulfated moieties (Fig. 1*A*). Functionally, the sulfated regions of HS provide binding sites for growth factors to prevent their degradation, act as receptors for proteases and protease inhibitors, facilitate cell-to-cell interactions, and can form ternary complexes with tyrosine kinase-type growth factor receptors to impact cell signaling (Fig. 1*A*) (2). While most of the enzymes responsible for synthesizing HS have been studied extensively over the years, the mechanisms that dictate their tissue-specific expression and activity to biosynthesize HS chains with diverse structures and functions are unclear (3). Moreover, regulatory pathways that impact HS assembly and HS–protein interactions in disease states, such as cancer, are understudied despite their relevance in driving certain disease pathologies (4).

**Figure 1:**
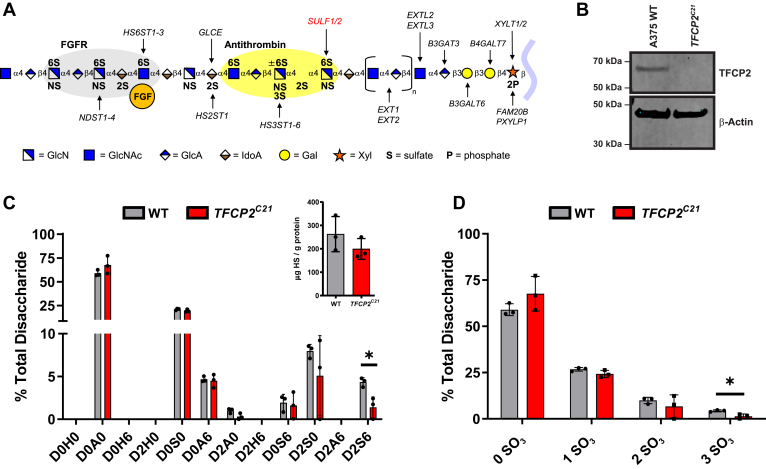
**TFCP2 regulates heparan sulfate assembly.***A*, Diagram of heparan sulfate (HS) biosynthesis. HS assembles while attached *via* a tetrasaccharide to a proteoglycan core protein. Secreted endosulfatases (SULF1/SULF2) are involved in the removal of 6-O sulfate groups. Figure adapted from (66). *B*, Western blot for CRISPR/Cas9 inactivation of TFCP2 in A375 human melanoma cells. Beta-actin (β-Actin) was used as a loading control. *C*, LC-MS quantification of disaccharides from HS in wild-type and *TFCP2*^*C21*^ cells (*t* test, n = 3, ∗*p* < 0.05). The absolute values for the disaccharides and the different classes of disaccharides are shown in Table S1. The disaccharide structure code is described in Table S1 and (67). (*inset*) LC-MS quantification of total HS in wild-type and *TFCP2*^*C21*^ cells (*t* test, n = 3, ∗*p* < 0.05). *D*, Sulfate groups (SO_3_) per disaccharide for HS isolated from wildtype and TFCP2 knockout cells (*t* test, n = 3, ∗*p* < 0.05). TFCP2, transcription factor cellular promoter 2.

Recent studies have begun to reveal that HS composition, its binding properties, and resultant biological activity depends on diverse factors in addition to the catalytic properties of the biosynthetic enzymes. Multiple groups have found transcriptional regulatory elements (5, 6, 7), 5′UTR and 3′UTR sequences (8), Golgi localized partners (9), and alternate splice variants (10) that can tune HS assembly. We and others have also begun to utilize functional genomics and bioinformatic tools to search across the genome to identify transcriptional (11, 12) and epigenetic (13, 14) regulatory mechanisms. We recently performed genome-wide CRISPR screens in human cancer cells and identified novel pathways involved in HS assembly (14). In the current study, we focus on one of the top hits identified from these screens, the alpha-globin transcription factor TFCP2, which has not been previously investigated for its role in regulating heparan sulfate biosynthesis.

Transcription factor cellular promoter 2, late SV40 factor, CP2 (TFCP2) is a member of a subfamily of Grainyhead-like (GRHL) transcription factors and is ubiquitously expressed in all human cells. It was originally discovered to bind and activate the alpha-globin promoter in erythroid cells (15). The gene responsible for encoding *TFCP2* is located on chromosome 12 and has three alternatively spliced mRNAs. TFCP2 has an N-terminal DNA binding domain and a C-terminal domain that is involved in its dimerization. It exists as a dimer in solution but forms a tetramer when bound to DNA (16). TFCP2 is known to be important in reproduction, cell cycle, hematopoiesis, expression of Human Immunodeficiency Virus (HIV) genes, and the development of cancer (17). Interestingly, targeted disruption of *Tfcp2* in mice gave no detectable phenotype, which may be the result of compensation *in vivo* by other related transcription factors (*e.g.*, UBP1) (18). TFCP2 has been studied most extensively in hepatocellular carcinoma (HCC) where it is overexpressed in 90% of HCC cases and correlates with disease progression (19). TFCP2 functions as a co-factor for YAP-dependent transcription in liver malignancy (20) and pharmacological targeting of this factor in HCC leads to decreased tumor growth in mice (21). TFCP2 has also been implicated in pancreatic cancer progression (22), oral squamous cell carcinoma (23), colorectal cancer (24), and others (25). Interestingly, transgenic overexpression of TFCP2 in melanoma was shown to impede tumor growth (26). While TFCP2 remains an interesting target for cancer, the underlying molecular mechanisms of its role in tumorigenesis are uncertain.

In the present study, we investigated the regulatory role of TFCP2 in HS assembly by targeting this factor in A375 human melanoma cells. Structural analysis of HS, ligand binding experiments, RNA sequencing, and growth assays in TFCP2 knockout cells revealed that TFCP2 transcriptionally regulates HS assembly and HS–protein interactions *via* repression of sulfatase 1 (*SULF1*) expression. These results identify a unique regulatory role of TFCP2 in mammalian glycosylation and may provide a new target for human melanoma.

## Results

### TFCP2 regulates HS assembly and growth factor binding

In previous studies, genome-wide CRISPR/Cas9 screening assays in A375 human melanoma cells identified the alpha-globin transcription factor, TFCP2, as a potential regulator of HS assembly (14). TFCP2 is a highly conserved transcription factor ubiquitously expressed across all cell and tissue types (15) and has not previously been implicated in HS assembly. To investigate TFCP2 as a novel regulatory factor, we targeted *TFCP2* in A375 cells *via* CRISPR/Cas9 and generated a knockout cell line (hereafter referred to as *TFCP2*^*C21*^), which was confirmed by Western blotting (Fig. 1*B*) and sanger sequencing (Fig. S1*A*). We noticed no morphological changes in knockout cells compared to the wild-type parental line (Fig. S1, *B* and *C*). To investigate alterations in HS biosynthesis, we isolated cell surface HS from wild-type and *TFCP2*^*C21*^ cells by trypsin-digestion and anion-exchange chromatography. The samples were subsequently depolymerized into disaccharides using heparin lyases and aniline-tagged by reductive amination. Disaccharide analysis was performed by ion-pairing reverse-phase chromatography and quantitative high-resolution MS with mass standards as previously reported (27). *TFCP2*^*C21*^ cells showed a similar disaccharide profile to wild-type cells except for a significant decrease in trisulfated disaccharides (D2S6) (Fig. 1, *C* and *D* and Tables S1–S3). Interestingly, overall HS levels were not significantly altered between the genotypes (Fig. 1*C*, inset). These results suggested distinct changes in HS sulfation patterning, particularly highly sulfated domains, which are known to dictate specific HS–protein interactions (2).

**Figure 2:**
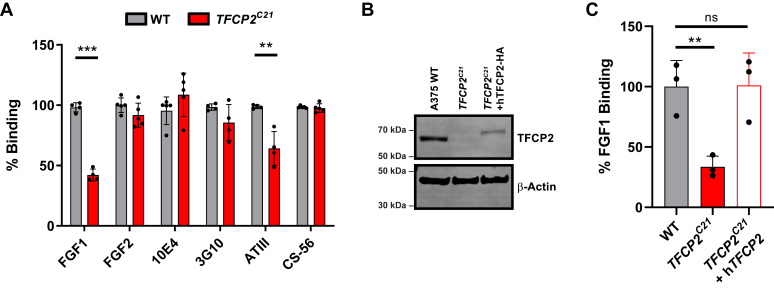
**Cell surface binding and TFCP2 rescue experiments.***A*, *TFCP2*^*C21*^ cells show a significant decrease in binding of a subset of protein ligands by flow cytometry (n ≥ 4, *t* test, ∗∗*p* < 0.01, ∗∗∗*p* < 0.001). *B*, Transfection of an HA-tagged human TFCP2 cDNA cassette restored TFCP2 expression in *TFCP2*^*C21*^ cells. *C*, Reintroduction of TFCP2 restored fibroblast growth factor 1 (FGF1) binding to wildtype levels (n = 3, *t* test, ∗∗*p* < 0.01). TFCP2, transcription factor cellular promoter 2.

To investigate whether changes in the HS assembly of *TFCP2*^*C21*^ cells could impact HS-protein interactions at the cell surface, we measured the binding of a collection of known HS-binding ligands to wild-type and *TFCP2*^*C21*^ cells by flow cytometry (Fig. 2*A*). From this analysis, we found a significant decrease in fibroblast growth factor 1 (FGF1) binding in *TFCP2*^*C21*^ cells, whereas there was no significant change in fibroblast growth factor 2 (FGF2) binding, each of which requires distinct sulfation patterns for interaction with HS on the cell surface (28, 29, 30). We also detected a decrease in antithrombin binding, which binds to specific 3-*O*-sulfated pentasaccharide subunits of heparin and HS (Fig. 1*A*) (31). Moreover, we found no difference in the binding of an antibody that recognizes *N*-sulfated/*N*-acetylated hybrid regions of HS (10E4) (32), consistent with the lack of any change in *N*-sulfated disaccharides (Fig. 1*C*). In addition, no difference was detected in the binding of an antibody that detects an HS neoepitope generated by heparin lyase digestion (3G10), indicating no change in the number of HS chains attached to cell surface proteoglycans (33). We also analyzed the expression of a related subtype of GAGs on the cell surface, chondroitin sulfate (CS), using the anti-CS antibody, CS-56 (34). We detected no change in CS-56 binding in mutant cells compared to wildtype, suggesting a specific alteration to HS synthesis in *TFCP2*^*C21*^ cells.

To corroborate our findings, we generated an additional TFCP2 knockout clone (*TFCP2*^*C9*^), which gave a similar binding profile to *TFCP2*^*C21*^ (Fig. S2, *A* and *B*). Additionally, we wanted to confirm that the phenotype we observed was due to the absence of TFCP2 and not an off-target effect of the CRISPR/Cas9 system. Therefore, we performed a rescue experiment where we transfected an expression plasmid containing HA-tagged *TFCP2* cDNA into *TFCP2*^*C21*^ cells (Fig. 2*B*). We observed a restoration of FGF1 binding in the rescued *TFCP2*^*C21*^ cells back to A375 wild-type levels (Fig. 2*C*).

### Transcriptome profiling of TFCP2 knockout cells

**Figure 3:**
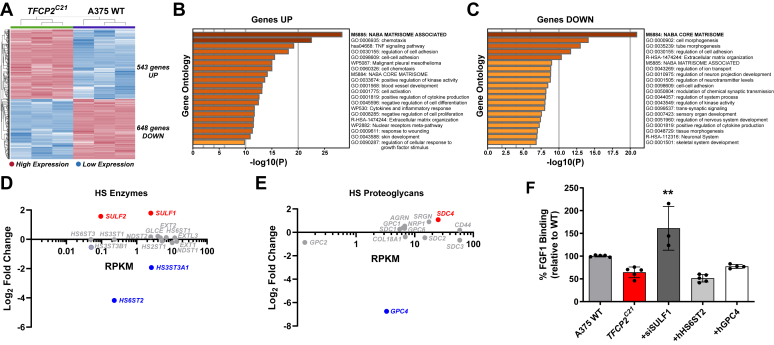
**Transcriptome analysis of A375 TFCP2 knockout cells.***A*, Heat map illustrating RNA-Seq differential expression data of genes upregulated (*red*) and downregulated (*blue*) in *TFCP2*^*C21*^ cells compared to A375 wild-type cells. Gene set enrichment analysis of significantly (*B*) upregulated (log2 ≥ 1, *p* ≤ 0.05) and (*C*) downregulated (log2 ≤ −1, *p* ≤ 0.05) genes in A375 wildtype and *TFCP2*^*C21*^ RNA-Seq datasets (n = 3). mRNA expression of (*D*) HS enzymes and (*E*) proteoglycans from RNA-seq data. Top differentially expressed genes (log2 ± 1, *p* ≤ 0.05) are shown in *red* and *blue*, respectively. *F*, FGF1 binding of *TFCP2*^*C21*^ cells transfected with indicated expression plasmids (hHS6ST2 or hGPC4) or an siRNA targeting *SULF1* compared to mock-transfected wildtype cells (*t* test, n ≥ 3, ∗∗*p* < 0.01). HS, Heparan sulfate; SULF1, sulfatase 1; TFCP2, transcription factor cellular promoter 2.

Since TFCP2 is a known transcriptional regulator (17) and we observed significant changes in HS structure and HS-protein interactions, we performed RNA sequencing on wildtype and *TFCP2*^*C21*^ to compare their transcriptome profiles (Fig. 3). A heat map of global gene expression changes showed a distinct gene expression signature for the *TFCP2*^*C21*^ knockout cells, with a subset of 543 genes upregulated and 648 genes downregulated (Fig. 3*A*). Gene ontology analysis of differentially expressed gene sets revealed that a majority of these genes were grouped as factors related to the extracellular matrix (*Core Matrisome, Matrisome-associated, Extracellular Matrix Organization*) (Fig. 3, *B* and *C*). These results suggest that TFCP2 may be a major regulator of the components of the extracellular matrix, which is consistent with previous reports showing that TFCP2 controls the expression of matrix proteins including fibronectin (35), osteopontin (19), and matrix metalloprotease-9 (36). When we investigated expression changes in HS biosynthetic machinery and modifying enzymes in our dataset, we found that both 6-*O*-endosulfatases, *SULF1* and *SULF2*, were significantly upregulated in *TFCP2*^*C21*^ cells while expression of the HS 6-*O*-sulfotransferase *HS6ST2* and 3-*O*-sulfotransferase *HS3ST3A1* were reduced (Fig. 3*D*). We also investigated expression changes in HS-associated proteoglycans (PGs). We found slight changes in expression across various cell surface PGs with the only large significant decrease in glypican-4 (*GPC4*) expression, a glycosylphosphatidylinositol-anchored proteoglycan (Fig. 3*E*) (37). Overall, these results correlated well with the structural analysis of cell surface HS (Fig. 1, *C* and *D*) and ligand binding assays where we observed a decrease in the FGF1 and ATIII binding (Fig. 2*A*). Importantly, we did not detect a significant decrease in the expression of FGF receptors (*FGFR1-4*), which may have explained the decrease in FGF1 binding we observed in the TFCP2 knockout cells since FGF1 can bind to and activate all FGFRs in conjunction with HSPGs (38). Conversely, we found significant upregulation of specific FGFRs (*FGFR1* and *FGFR3*), thus supporting that alterations in HS assembly cause the decrease in FGF1 binding at the cell surface (see Table S5).

### TFCP2 is a repressor of *SULF1* expression

**Figure 4:**
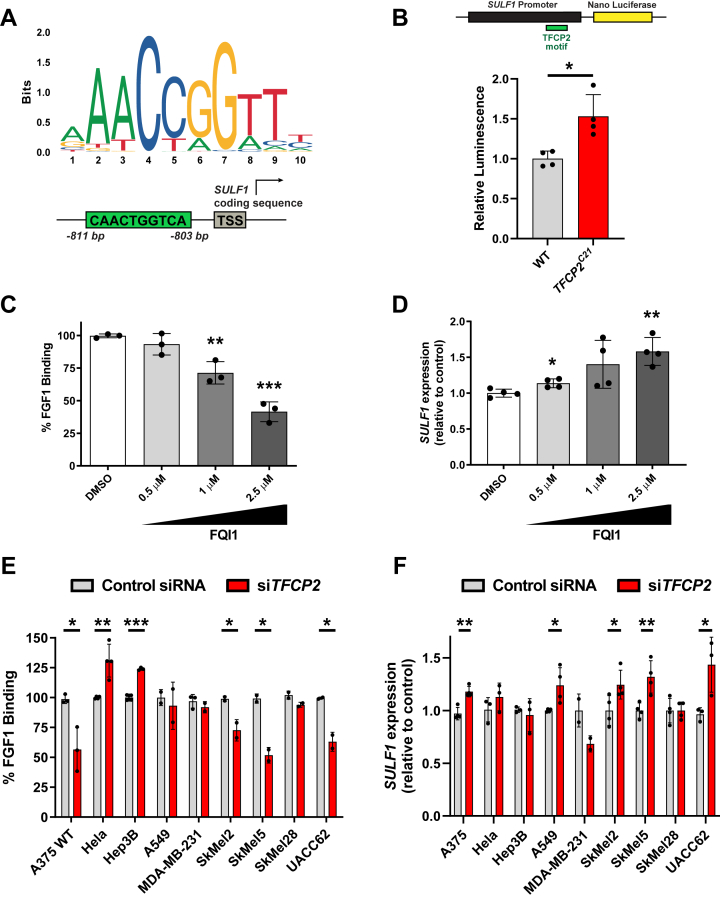
**TFCP2 regulates SULF1 expression.***A*, TFCP2 binding motif is found in the promoter of human *SULF1*. *B*, *SULF1* promoter drives expression of nano luciferase (NLuc) and is increased in *TFCP2*^*C21*^ cells (*t* test, n = 4, ∗*p* < 0.05). The TFCP2 inhibitor, factor quinolinone inhibitor 1 (FQI1), (*C*) reduces FGF1 binding and (*D*) increases *SULF1* expression dose dependently in A375 wild-type cells (*t* test, n ≥ 3, ∗*p* < 0.05, ∗∗*p* < 0.01, ∗∗∗*p* < 0.001). *E*, FGF1 binding is decreased in human melanoma cells compared to other human cancer lines (*t* test, n ≥ 2, ∗*p* < 0.05, ∗∗*p* < 0.01, ∗∗∗*p* < 0.001). *F*, *SULF1* expression is upregulated upon knockdown of *TFCP2* expression in human melanoma cells (*t* test, n ≥ 2, ∗*p* < 0.05, ∗∗*p* < 0.01). HS, Heparan sulfate; SULF1, sulfatase 1; TFCP2, transcription factor cellular promoter 2.

To connect changes in HS gene expression with TFCP2 regulation of HS assembly and ligand binding, we performed targeted rescue experiments by restoring the expression of differentially expressed HS genes in *TFCP2*^*C21*^ cells. We focused on SULF1 and HS6ST2 due to previous studies showing that FGF1 binding is dependent on HS 6-*O* sulfation (29, 39, 40). Despite being upregulated in *TFCP2*^*C21*^ cells, multiple attempts to measure *SULF2* expression in wild-type and TFCP2 knockout cells *via* quantitative PCR were unsuccessful, most likely due to very low expression (Table S5). Knockdown of *SULF1* in the *TFCP2*^*C21*^ cells gave a significant restoration of FGF1 binding compared to mock-transfected *TFCP2*^*C21*^ cells, while overexpression of *HS6ST2* cDNA gave no detectable change in FGF1 binding (Figs. 3*F* and S3). To confirm that changes in HSPG expression were not driving the FGF1 phenotype, we also overexpressed *GPC4* in TFCP2 knockout cells and found no significant change in FGF1 binding. Since the knockdown of *SULF1* expression restored FGF1 binding in *TFCP2*^*C21*^ cells, we subsequently examined whether TFCP2 functions as a direct regulator of *SULF1* expression in A375 cells. Upon scanning the *SULF1* promoter region for TFCP2 putative binding sites using the JASPAR database and scan function (41), we identified a TFCP2 binding sequence −803 bp from the transcription start site (Fig. 4*A*). To confirm its functionality, we constructed a gene expression cassette with the *SULF1* promoter controlling a nano-luciferase reporter gene and found a significant increase in luciferase expression in the absence of TFCP2, corroborating negative regulation of *SULF1* by TFCP2 through direct binding to its promoter (Fig. 4*B*).

As an orthogonal approach to confirm these findings, we took advantage of a previously published pharmacological inhibitor of TFCP2, factor quinolinone inhibitor 1 (FQI1) (Fig. S4*A*), which was shown to specifically block TFCP2 DNA binding activity and inhibited tumor growth in hepatocellular carcinoma mouse xenografts (21). A375 wild-type cells were treated with increasing concentrations of FQI1 (0–2.5 μM) for 24 h and assessed for FGF1 binding and *SULF1* expression. We found a dose-dependent decrease in FGF1 binding (Fig. 4*C*) and a stepwise increase in *SULF1* expression, as measured by quantitative PCR (Fig. 4*D*), with no changes in TFCP2 protein levels (Fig. S4*B*). Finally, we were interested in whether the regulation of HS assembly by TFCP2 was conserved across various human cell types. Therefore, we knocked down *TFCP2* expression using RNA interference in A375 (melanoma), HeLa (cervical adenocarcinoma), A549 (lung carcinoma), Hep3B (liver hepatocellular carcinoma), and MDA-MB-231 (triple negative breast adenocarcinoma) cells (Fig. S4) and measured FGF1 binding and *SULF1* expression. Interestingly, we observed a similar decrease in FGF1 binding in A375 cells, but we found no significant change in A549 and MDA-MB-231 cells and a slight increase in binding in HeLa and Hep3B cells (Fig. 4*E*). Subsequently, we measured *SULF1* expression by quantitative PCR and found an increase in *SULF1* mRNA levels in A375 and A549 cells (Fig. 4*F*).

To support whether the FGF1 binding defect was specific to human melanoma cells, we knocked down *TFCP2* expression in a panel of patient-derived malignant melanoma tumor lines with diverse genotypes (SkMel2, SkMel5, SkMel28, and UACC62). We found a similar decrease in FGF1 binding in most of these lines, except for SkMel28 cells (Fig. 4*E*), which uniquely harbors a homozygous mutation in epidermal growth factor receptor (EGFR) in addition to mutant B-Raf (V600E) (42). Correspondingly, we found an increase in *SULF1* expression in SKMel2, SkMel5, and UACC62 melanoma cells, with no change in *SULF1* mRNA levels in SKMel28 cells (Fig. 4*F*). Altogether, these results suggest that TFCP2 may play a unique HS regulatory role in melanoma.

### *TFCP2* inactivation impedes SULF1-dependent melanoma cell growth

**Figure 5:**
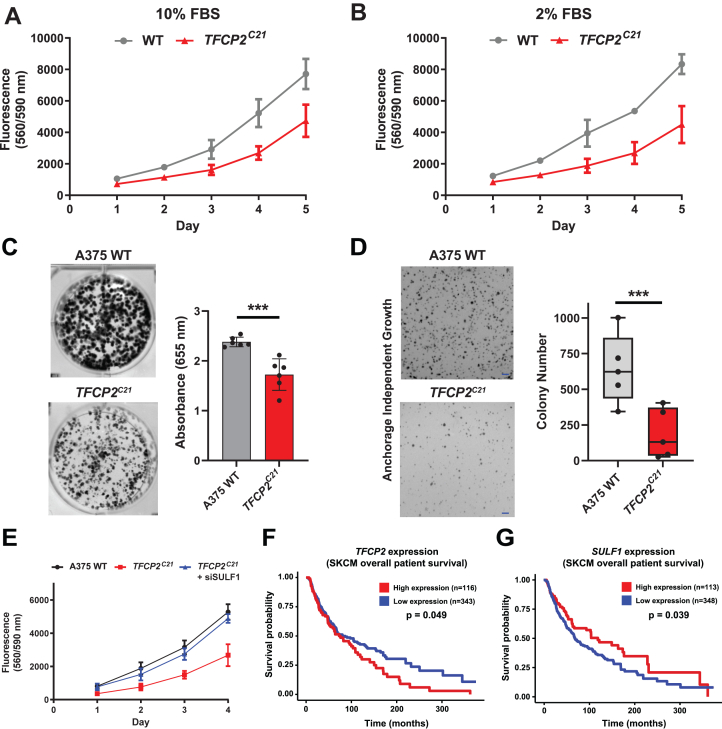
**TFCP2 inactivation impedes SULF1-dependent melanoma cell growth.***TFCP2*^*C21*^ cells exhibited decreased cell growth over 5 days under (*A*) standard (10% FBS) and (*B*) low serum (2% FBS) conditions as measured by Cell Titer Blue (Two-way ANOVA; n = 3, ∗∗*p* < 0.01). *C*, Clonogenic assays under normal growth conditions. After 14 days, colony growth was quantified by methylene blue staining and absorption readings at 655 nm (*t* test, n = 6, ∗∗∗*p* < 0.001). *D*, Anchorage-independent growth was analyzed by soft agar assays. Colony number was measured after 21 days incubation. Data are shown as mean colony number of triplicate wells from five independent experiments (*t* test, n = 5, ∗∗∗*p* < 0.001). Scale Bar = 1 mm. *E*, Knockdown of *SULF1* in TFCP2 knockout cells restores cell growth to wildtype levels (Two-way ANOVA; n = 3, ∗∗∗*p* < 0.001). Association of overall survival with (*F*) *TFCP2* and (*G*) *SULF1* expression levels in the TCGA skin cutaneous melanoma (SKCM) cohort (*p* values from the log-rank test). HS, Heparan sulfate; SULF1, sulfatase 1; TFCP2, transcription factor cellular promoter 2.

HS is known to play an essential role in cell signaling and growth and has also been implicated in tumorigenesis across a variety of cancers, particularly due to its role in fibroblast growth factor binding at the cell surface and cell signaling (43). HS has specifically been shown to play a key role in melanoma development and progression (44) and, more recently, melanoma resistance mechanisms (45). Additionally, TFCP2 has been implicated in cancer progression (25) and may play a role in melanoma growth (26). Therefore, we were interested in whether targeting TFCP2 alters cell growth. *TFCP2*^*C21*^ cells exhibited a significant growth defect in complete media (10% fetal bovine serum, FBS) as well as in starved conditions (2% FBS) compared to wild-type cells (Fig. 5, *A* and *B*). Additionally, TFCP2 knockout cells did not form colonies as readily in clonogenic growth assays (Figs. 5*C* and S2, *C* and *D*) and in anchorage-independent growth in soft agar (Fig. 5*D*). To investigate whether SULF1 is important for cell growth in *TFCP2*^*C21*^ cells, we knocked down *SULF1* expression in *TFCP2*^*C21*^ cells. We observed full restoration of cell growth in *SULF1*-targeted *TFCP2*^*C21*^ cells (Fig. 5*E*). These findings indicate that TFCP2 regulates the proliferation of A375 melanoma cells and that this regulation occurs *via* repression of *SULF1* expression. To determine whether the expression of TFCP2 and/or SULF1 correlate with the survival of patients with melanoma, we analyzed clinical data from the TCGA database using the University of Alabama at Birmingham UALCAN interactive web portal (46). Interestingly, high TFCP2 expression (*p* = 0.049) and low SULF1 expression (*p* = 0.039) significantly correlated with poorer overall survival outcomes for patients with skin cutaneous melanoma (Fig. 5, *F* and *G*). Overall, our results, as well as clinical patient data, suggest that TFCP2 and SULF1 should be further explored as potential targets for melanoma therapy.

## Discussion

HS polysaccharides are key components of the extracellular matrix and interact with many key ligands at the cell surface, including fibroblast growth factors, which implicates them as key effectors in many important cellular processes, including cell proliferation, signaling, development, and angiogenesis (2). Despite their important role in cell homeostasis, the molecular mechanisms governing the spatiotemporal regulation of HS assembly are largely unknown (3). In the current study, we investigated a top hit from recent CRISPR/Cas9 screens (14), the alpha-globin transcription factor TFCP2, to investigate its role in regulating HS assembly in human cancer cells. Historically, TFCP2 has been studied primarily in the context of development (47) and as a pro-oncogene in human cancers (25), yet little is known about its role in controlling glycosylation.

In this study, CRISPR/Cas9 targeting of *TFCP2* in human melanoma cells led to distinct changes in HS gene expression and growth factor binding at the cell surface. Interestingly, TFCP2 knockout cells did not exhibit global changes in HS amount; instead, we discovered changes in the fine structure of cell surface HS, with a reduction in trisulfated (D2S6) disaccharide subunits (Fig. 1). These highly sulfated subunits are enriched in sulfated domains that provide binding sites for ligands at the cell surface and are the preferred molecular substrate for the secreted sulfatases, SULF1 and SULF2 (48). Cell surface binding assays revealed a selective decrease in FGF1 binding to *TFCP2*^*C21*^ cells that was rescued upon transgenic expression of a TFCP2 cDNA construct (Fig. 2). Additionally, we observed a decrease in antithrombin binding, which requires specific 3-*O* and 6-*O* sulfated sites (49, 50). Together, these results indicate that TFCP2 plays a role in the regulation of HS fine structure and specific HS-protein interactions, the organization of which is important for development and tumor progression (51, 52). Intriguingly, we found that knockdown of TFCP2 in human cancer cell lines from other tissues (lung, breast, liver, and cervix) did not give a similar phenotype, suggesting that TFCP2 may regulate HS assembly in a tissue- or cell-specific manner. This finding was strengthened when we saw a similar reduction in FGF1 binding in cells isolated from other melanoma patients with diverse genotypes (Fig. 4*E*).

Transcriptomic analysis of *TFCP2*^*C21*^ cells *versus* wild-type cells showed a distinct expression signature, with the majority of differentially expressed genes categorized as extracellular matrix factors (Fig. 3). These results are in agreement with previous reports where TFCP2 was found to directly regulate the expression of ECM proteins, including fibronectin (35) and matrix metalloprotease-9 (36), both of which are known to interact with HSPGs (53, 54). From our analysis, we found up- and downregulation of multiple genes encoding for enzymes involved in HS assembly (*HS6ST2, HS3ST3A1, SULF1, SULF2*), which is in line with recent reports describing TFCP2’s role as both a transcriptional activator and repressor (16). Our targeted rescue experiments indicated that upregulation of SULF1 drove the observed decrease in FGF1 binding (Fig. 3*F*), most likely due to the removal of key 6-*O*-sulfated sites in highly sulfated domains of HS at the cell surface (48). Luciferase assays revealed direct TFCP2-mediated repression of *SULF1* expression (Fig. 4*B*), which could be caused *via* the recruitment of factors involved in DNA methylation (55) or chromatin remodeling (56). Intriguingly, targeting another top hit from our published CRISPR screen in A375 cells, the histone demethylase and polycomb repressive complex (PRC1) member KDM2B, resulted in a similar differential HS gene expression profile to TFCP2 knockout cells, including upregulation of *SULF1* (14). A recent study also described the epigenetic repression of SULF1 in chondrosarcoma by the histone methyltransferase, EZH2 (57). These studies suggest there may be coordinated epigenetic and/or transcriptional regulatory networks controlling sulfatase expression and HS composition in distinct cell types and disease states.

Interestingly, the inactivation of TFCP2 in A375 cells reduced their 2D and 3D growth (Fig. 5), which is contrary to a previous study showing that overexpression of TFCP2 inhibits melanoma cell growth (26). This earlier report found that transient overexpression of TFCP2 in mouse melanoma cells gave a decrease in 3D growth and tumor growth *in vivo*, respectively. It is possible that overexpression of TFCP2 could result in dominant-negative effects to cause a novel phenotype (58), or TFCP2 regulation could be diverse among different species and/or cell types. TCGA patient survival data indicate that high TFCP2 and low SULF1 expression leads to lower survival rates (Fig. 5, *F* and *G*). TFCP2-mediated repression of SULF1 may link these observations, and our data showing that knockdown of *SULF1* increases melanoma cell growth (Fig. 5*E*) correlate well with these findings. Previous studies have shown that overexpression of SULF1 in melanoma inhibits cell growth and tumor growth in mice (59). Broadly, SULF1 is epigenetically silenced in many cancer types (60, 61, 62) and forced expression decreases tumor cell proliferation, migration, and invasion (63). This is not surprising due to the inhibitory role of SULF1 in growth factor and cytokine signaling *via* the reduction of sulfation of cell surface HS. Future studies will be focused on investigating the TFCP2/SULF1 regulatory axis as a viable drug target in melanoma and other tumor types.

## Experimental procedures

### Cell culture

A375 (ATCC CRL-1619), HEK293T (ATCC CRL-3216), SkMel2 (ATCC HTB-68), SkMel5 (ATCC HTB-70), SkMel28 (ATCC HTB-72), UACC62 (CVCL_1780), A549 (ATCC CCL-185), and MDA-MB-231 (ATCC HTB-26) cells were grown in Dulbecco's Modified Eagle Medium (DMEM; Gibco) supplemented with 10% (v/v) FBS and 1% (v/v) penicillin/streptomycin at 37 °C under an atmosphere of 5% CO_2_/95% air. HeLa (ATCC CCL-2) and Hep3B (ATCC HB-8064) cells were grown in Minimal Essential Media (Gibco) supplemented with 10% (v/v) FBS and 1% (v/v) penicillin/streptomycin at 37 °C under an atmosphere of 5% CO_2_/95% air. Cells were sub-cultured every 3 to 4 days and were revived from liquid nitrogen after ≤10 passages. All transfected cell lines were cloned and stored in liquid nitrogen.

### Cell line generation

HEK293T cells were co-transfected with Fugene 6 (Promega) and 4 μg each of a viral envelope plasmid (pMD2.g, a gift from Didier Trono and purchased from Addgene, #12259), packaging plasmid (psPAX2, a gift from Didier Trono and purchased from Addgene, #12260), and Cas9 expression plasmid (lentiCas9-Blast, a gift from Feng Zhang and purchased from Addgene, #52962) to generate Cas9 lentiviral particles. Lentiviral particles were collected and added to A375 cells followed by selection with 2 μg/ml blasticidin to generate Cas9-expressing cells. A375 TFCP2 mutant cell lines were generated by ligation of sgRNAs targeting human *TFCP2* (5′- GTGCTGGTGCCTATAGCATG -3′) into the lentiGuide-Puro vector (a gift from Feng Zhang and purchased from Addgene, #52963) and co-transfection with Fugene six into HEK293T cells, along with viral plasmids pMD2.g and psPAX2, to generate lentiviral particles, which were subsequently used to transduce Cas9-expressing A375 cells. After selection of the transduced cell pool with puromycin (1 μg/ml) for 3 days, surviving cells were seeded onto a 96-well plate by limiting dilution and clonal populations were established.

### Growth experiments and soft agar assays

For growth curves, A375 wild-type, *TFCP2*^*C21*^, or siRNA-transfected cells were plated in 96-well plates (1000 cells per well) in complete media containing 10% or 2% FBS. Cell viability was measured with Cell Titer Blue (Promega) every 24 h for 4 to 5 days. For clonogenic assays, colony formation was assessed by seeding cells at a density of 1000 cells per well in 6-well plates and incubating for 14 days in DMEM with 10% FBS and 1% penicillin/streptomycin in an atmosphere of 5% CO_2_ at 37 °C. Complete media was supplemented every 5 days. Colonies were visualized with methylene blue (12.5 mM in methanol) for 10 min at room temperature, washed with deionized water, and imaged. For quantification, methylene blue was dissolved in 0.7 M sodium citrate in 50% ethanol and absorbance was measured at 655 nm.

For soft agar assays, 7500 cells/well were plated in 6-well plates in complete DMEM containing 0.3% agarose, with a 0.6% agarose underlay. The colonies were grown for 21 days and supplemented with four drops of DMEM every 4 days. The colonies were stained with 0.01% (w/v in 10% ethanol) crystal violet, imaged, and scored using the “Analyze Particles” procedure in ImageJ software.

### Rescue experiments and siRNA transfections

Lentiviral particles carrying the human *TFCP2* gene were produced by co-transfection of HEK293T cells with a psPAX2 packaging plasmid (Addgene plasmid #12260), the VSV-G-encoding plasmid pMD2.g (Addgene plasmid #12259), and a human TFCP2 lentiviral vector (pLenti-GIII-CMV-TFCP2-HA, Applied Biological Materials). Medium containing the lentivirus particles was collected and used to infect A375 TFCP2 mutant cells. After infection, the cells were cultured with 2 μg/ml puromycin to select for stably transduced cells.

For A375 rescue experiments, *TFCP2*^*C21*^ cells (2 × 10^5^ cells/well) were transfected with Lipofectamine LTX with Plus reagent (Invitrogen) and a human HS6ST2-HA expression plasmid (Applied Biological Materials Inc), a human GPC4-HA expression plasmid (Sino Biological), or an siRNA targeting human *SULF1* (SASI_Hs02_00330796, Sigma). For knockdown experiments, cells (2 × 10^5^ cells per well) were transfected with Lipofectamine LTX with Plus reagent (Invitrogen) and a siRNA targeting human *TFCP2* (SASI_Hs01_00128372; Sigma) according to the manufacturer’s instructions. Cells were incubated with this mixture for 4 h, after which the medium was replaced with DMEM or MEM (+ 10% FBS). FACS binding and qPCR experiments were performed 48 h post-transfection.

### RNA extraction and quantitative PCR

RNA was isolated from cell lines and transfected cells using TRIzol (Invitrogen) and the RNeasy Kit (Qiagen) following the manufacturer’s instructions. cDNA was prepared from total RNA using the SuperScript IV First Strand Synthesis kit (Invitrogen) using random hexamers following the manufacturer’s instructions. qPCR was performed using cDNA and SYBR Green Master Mix (Applied Biosystems) following the manufacturer’s instructions. The expression of *YWHAZ* (housekeeping gene) was used to normalize the expression of target genes between samples. The primers used for quantitative PCR are provided in Table S4.

### RNA sequencing and differential gene expression analysis

Total RNA extracted from wildtype and knockout cell lines was submitted for library preparation and next-generation sequencing (HudsonAlpha Discovery). Raw RNA sequencing data was analyzed using the GeneGlobe RNA-seq Analysis Portal (Qiagen). Adapter sequences and low-quality bases were trimmed and sequence alignment was performed against the Human genome (GRCH.38; GCF_000001405.38) with the default parameters. Differential gene expression analysis was performed using this tool (Table S5). After Benjamini-Hochberg FDR correction, genes with adjusted *P*-values ≤0.05 and fold change ±2 were considered as differentially expressed genes (DEGs). Functional annotation and gene set enrichment analysis of the top differentially expressed genes were carried out using Metascape (http://metascape.org/) (64).

### Western blotting

Total protein was extracted from cells using RIPA Lysis and Extraction Buffer (EMD Millipore) supplemented with protease inhibitors (Roche). Protein concentration was determined by BCA assay (Thermo Scientific-Pierce). Protein samples were subjected to SDS-polyacrylamide gel electrophoresis (PAGE) (4–12% Bis-Tris, Invitrogen), blotted on polyvinylidene difluoride membranes (Invitrogen), and probed for TFCP2 (rabbit anti-TFCP2, Cell signaling technology #80784, 1:1000) and β-actin (mouse anti-β-Actin, Cell Signaling Technology #3700, 1:1000). Membranes were blocked with 5% milk in Tris-buffered saline and 0.1% Tween for 1 h at room temperature than were incubated with the respective primary antibodies in 5% milk in Tris-buffered saline (+0.1% Tween) at 4 °C overnight. Mouse and rabbit primary antibodies were incubated with secondary Odyssey IR dye antibodies (1:14,000; LI-COR Biosciences) and visualized with an Odyssey IR imaging system (LI-COR Biosciences).

### Protein biotinylation

Heparin-Sepharose (100 μl, Cytiva) was pre-equilibrated with PBS (Gibco) and then loaded with human FGF1 (Peprotech, #100-17A) or human FGF2 (Peprotech, #100-18B) dissolved in PBS, as previously described (65). The flow-through was reloaded onto the column twice to ensure complete binding. After washing twice with PBS, a 0.6 mg/ml solution of Sulfo-NHS-LC-biotin (Thermo Fisher) in PBS was loaded onto the column and incubated for 1 h at room temperature. Each column was washed three times with PBS, then bound biotinylated protein was eluted with 0.4 ml of PBS buffer containing an additional 2 M NaCl. All biotinylated proteins were stored at −80 °C.

### Flow cytometry

Cells grown in monolayer culture were washed with PBS, lifted using 10 mM EDTA in PBS, and incubated in suspension for 30 min at 4 °C with 0.5 μg/ml mAb 10E4 (AMSBio #370255-1, Clone F58-10E4, 1:2000), 1 μg/ml mAb 3G10 (AMSBio, #370260-S, clone F69-3G10, 1:1000), 1 μg/ml mAb anti-chondroitin sulfate (Sigma #C8035, Clone CS-56, 1:2000), 80 nM biotin-FGF1, or 2.5 nM biotin-FGF2, respectively. For heparin lyase pre-treatment, lifted cells were incubated with 5 mU/ml each of heparin lyases I, II, and III (IBEX) for 30 min at 37 °C in PBS (+0.1% BSA). Alternatively, cells were incubated for 1 h at 4 °C with 500 nM human antithrombin (Anaira). Bound 10E4 and CS-56 were detected with 2 μg/ml anti-mouse IgM AlexaFluor 647 (Invitrogen, #A-21238, 1:1000). Bound 3G10 was detected with 2 μg/ml anti-mouse IgG AlexaFluor 488 (Invitrogen, # A11001, 1:1000). Binding of biotinylated proteins was detected by streptavidin-Cy5 (Molecular Probes, 1:1000). Bound antithrombin was detected with 2 μg/ml anti-AT pAb (R&D Systems, AF1267, 1:100) followed by 2.5 μg/ml donkey anti-goat conjugated to AlexaFluor 647 (Invitrogen, #A-21447, 1:1000). Flow cytometry was performed using a CytoFLEX S (Beckman Coulter) flow cytometer (≥10,000 events/sample), and raw data were analyzed using FlowJo Analytical Software v10.8 (Becton Dickinson). Cells were gated according to forward and side scattering. The extent of protein binding was quantified using the geometric mean of the fluorescence intensity. These values were plotted and further analyzed using GraphPad Prism v9.0.

### HS purification and LC/MS analysis

Cells were seeded at 0.5 × 10^6^ cells/ml in a 10 cm plate and harvested when confluent. Briefly, cells were washed with PBS, lifted with trypsin (Gibco), and the trypsin-released glycosaminoglycans were digested with Pronase (0.5 mg/ml, Sigma) overnight at 37 °C. The product was filtered and passed through a DEAE-Sephacel (Cytiva) column equilibrated in 50 mM sodium acetate buffer, pH 6.0, containing 200 mM NaCl then passed through a PD-10 desalting column (Cytiva). The desalted product was then treated with DNase then passed through the DEAE-Sephacel and PD-10 columns a second time. For HS disaccharide analysis, lyophilized GAGs were incubated with 2 mU each of heparin lyases I, II, and III for 16 h at 37 °C in a buffer containing 40 mM ammonium acetate and 3.3 mM calcium acetate, pH 7. HS disaccharides were aniline-tagged and analyzed by RP-LC-MS on a LTQ XL Orbitrap mass spectrometer, as previously described (27).

### JASPAR motif analysis

Binding sequences of TFCP2 before the transcriptional start site of *SULF1* were predicted by JASPAR and sorted by "Relative Score". The motif for TFCP2 was identified in the JASPAR database (https://jaspar.genereg.net/), then a FASTA-formatted sequence ∼2000 bp upstream from the transcriptional start site (TSS) of *SULF1* was scanned with selected matrix models using the “Scan” tool. The relative profile score threshold was set at 80%.

### Luciferase assays

A 1.1 kb portion of the human *SULF1* promoter containing the predicted TFCP2 binding motif (as identified through the JASPAR database) was amplified (Fwd: TAAGCAAAGCTTAAACAATCCCCCTCCCAGT; Rev: TGCTTAAAGCTTTCAGCACAGTGGTGTGTCAA) and digested with HindIII and cloned into a NanoLuc luciferase vector, pNL1.1 (Promega). The resulting plasmid was co-transfected into A375 wild-type or *TFCP2*^*C21*^ cells with a firefly luciferase plasmid (pGL4.53, Promega) for normalization, and cell lysate was prepared and analyzed 48 h later (Nano-Glo Dual Luciferase Reporter Assay, Promega) on a Promega GloMax plate reader.

### Drug Treatments

Cells (2 × 10^5^ cells per well) were seeded in 6-well plates and treated with dimethyl sulfoxide (DMSO) or various concentrations of factor quinolinone inhibitor 1 (FQI1, Cayman Chemical Company). Cells were incubated for 24 h at 37 °C then analyzed *via* FACS, Western blot, or quantitative PCR, respectively, as described above.

### Correlation of gene expression from the TCGA database

For gene expression analysis of TCGA data, normalized data from the SKCM dataset were obtained from the UALCAN web portal (http://ualcan.path.uab.edu) (46). Survival plots were generated comparing high and low gene expression across the SKCM cohort.

### Statistics and Reproducibility

Statistical tests and sample sizes are indicated in the figure legends. ∗∗∗∗*p* < 0.0001; ∗∗∗*p* < 0.001; ∗∗*p* < 0.01; ∗*p* < 0.05. All tests were two-sided. Tests were performed in Prism v9.0 (GraphPad). Measurements were taken from distinct samples, and the number of biological replicates is indicated in the figure legends. Error bars represent mean ± standard deviation. Western blots were performed twice independently, and representative images are shown in the figures. Entire blot images can be found in the provided Source Data Figure.

## Data availability

Raw sequencing reads and results of the RNA sequencing analysis are available online at NCBI's Gene Expression Omnibus and are accessible through GEO Series accession number GSE224599 (https://www.ncbi.nlm.nih.gov/geo/query/acc.cgi?acc=GSE224599). Any additional data supporting the analyses in the manuscript are available from the corresponding author upon reasonable request.

## Supporting information

This article contains supporting information.

## Conflict of interest

The authors declare that they have no conflicts of interest with the contents of this article.

## Supporting information

Supporting Table S5

Supporting Figure S1 **A375 TFCP2 knockout clones.***A*, CRISPR sgRNA targeting of human *TFCP2* in A375 cells. Frameshift biallelic mutations in exon 1 were confirmed in clone 21 and clone 9 by Sanger sequencing (introns shown as *gray text*). Brightfield images of (*B*) A375 wildtype and (*C*) TFCP2 clone 21 show minimal differences in cell morphology. Scale Bar = 360 μm.

Supporting Figure S2 **A375 TFCP2 knockout clone C9 characterization.***A*, Western blot for TFCP2 knockout clone 9 in A375 cells. *B*, Ligand binding in A375 TFCP2 knockout clone C9 is comparable to clone C21 (*t*-tests; n ≥ 4, ∗*p* < 0.05, ∗∗∗*p* < 0.001, ∗∗∗∗*p* < 0.0001). *C* and *D*, TFCP2 knockout clone C9 exhibits reduced growth in clonogenic assays, as quantified by methylene blue staining and absorption readings at 655 nm (*t*-test, n = 3, ∗∗*p* < 0.01). Note: the A375 wildtype representative image shown is reused from Figure 5*C*, as clonogenic assays for both TFCP2 knockout clones were performed in parallel and compared to the same wildtype samples for each independent biological replicate experiment.

Supporting Figure S3 **Quantitative PCR data for rescue experiments.***A*, *SULF1* mRNA expression in A375 *TFCP2*^*C21*^ cells is restored to wildtype levels upon transfection of a silencing RNA targeting *SULF1* (siSULF1) (n = 2 independent experiments). *B*, Fold change in *HS6ST2* mRNA expression in A375 *TFCP2*^*C21*^ cells and in A375 *TFCP2*^*C21*^ cells expressing HS6ST2 cDNA from a lentiviral construct (hHS6ST2) (n = 2 independent experiments). *C*, Fold change in *GPC4* mRNA expression in A375 *TFCP2*^*C21*^ cells, and in A375 *TFCP2*^*C21*^ cells expressing GPC4 cDNA from an expression plasmid (hGPC4) (n = 2 independent experiments).

Supporting Figure S4 **Targeting TFCP2 in other human cell lines.***A*, Chemical structure for the TFCP2 inhibitor, factor quinolinone inhibitor 1 (FQI1). *B*, Western blot reveals no change in TFCP2 protein levels in A375 cells treated with various concentrations of FQI1 for 24 h. Western blot images show silencing of TFCP2 expression in (*C*) A375, (*D*) HeLa, (*E*) Hep3B, (*F*) A549, (*G*) SKMel5, (*H*) SKMel28, (*I*) UACC62 cell lines. Images are representative of two independent experiments. *J*, Quantitative PCR measuring *TFCP2* expression upon transfection of siRNA targeting *TFCP2* in human cell lines (n ≥ 2 independent experiments).

Source Data Figure Full western blot source images for (*A*) Figure 1*B*, (*B*) Figure 2*B*, (*C*) Fig S2*A*, (*D*) Fig S4*B*, (*E*–*I*) Fig S4, *A*–*G*.
