# Supplementary material for: TFCP2 is a transcriptional regulator of heparan sulfate assembly and melanoma cell growth
Source: J Biol Chem. 2023 Apr 13;299(6):104713. doi: 10.1016/j.jbc.2023.104713 (PMC10200990; doi:10.1016/j.jbc.2023.104713)
Supplement: Supporting Tables S1–S4 [file mmc1.docx]

## Supplementary Information

### Supplementary Table 1:

Disaccharide composition of A375 HS

| **Disaccharide Structure** | | **A375 Wildtype** | **A375 *TFCP2*^-/-^** |
| --- | --- | --- | --- |
| **Structure Code^a^** | **Unit Formula^b^** | **Abundance (% Total Disaccharide)^c^** | |
| D0H0 | ΔUA-GlcNH_2_ | – | – |
| D0A0 | ΔUA-GlcNAc | 59.0 ± 2.6 | 67.6 ± 7.6 |
| D0H6 | ΔUA-GlcNH_2_6S | – | – |
| D2H0 | ΔUA2S-GlcNH_2_ | – | – |
| D0S0 | ΔUA-GlcNS | 21.1 ± 0.8 | 19.5 ± 1.2 |
| D0A6 | ΔUA-GlcNAc6S | 4.7 ± 0.3 | 4.5 ± 0.6 |
| D2A0 | ΔUA2S-GlcNAc | 1.0 ± 0.2 | 0.3 ± 0.3 |
| D2H6 | ΔUA2S-GlcNH_2_6S | – | – |
| D0S6 | ΔUA-GlcNS6S | 1.9 ± 0.8 | 1.6 ± 1.3 |
| D2S0 | ΔUA2S-GlcNS | 8.0 ± 0.6 | 5.1 ± 3.8 |
| D2A6 | ΔUA2S-GlcAc6S | – | – |
| D2S6 | ΔUA2S-GlcNS6S | 4.3 ± 0.4 | 1.4 ± 1.0 |

^a^ The disaccharide structure code is described in (Lawrence, et al. Nat. Methods 2008)

^b^ ΔUA = 4,5-unsaturated uronic acid

^c^ –, not detected

### Supplementary Table 2:

Sulfation and *N*-substitution of glucosamine units

| **Unit** | **A375 Wildtype** | **A375 *TFCP2*^-/-^** |
| --- | --- | --- |
|  | **Constituents/100 disaccharides** | |
| Unsubstituted glucosamine | – | – |
| *N*-acetylglucosamine | 59 ± 3 | 68 ± 8 |
| *N*-sulfoglucosamine | 35 ± 3 | 28 ± 7 |
| Uronyl-2-*O*-sulfates | 13 ± 1 | 7 ± 5 |
| Glucosaminyl 6-*O*-sulfates | 11 ± 1 | 8 ± 3 |

### Supplementary Table 3:

Sulfate groups per disaccharide

| **# Sulfates** | **A375 Wildtype** | **A375 *TFCP2*^-/-^** |
| --- | --- | --- |
|  | **Abundance (% Total Disaccharide)** | |
| 0 SO_3_ | 59 ± 3 | 68 ± 8 |
| 1 SO_3_ | 27 ± 0.8 | 24 ± 2 |
| 2 SO_3_ | 10 ± 1 | 7 ± 5 |
| 3 SO_3_ | 4 ± 0.4 | 1 ± 1 |

### Supplementary Table 4:

qPCR Primer Sequences

| **Primer (Human)** | **Forward (5'-3')** | **Reverse (5'-3')** |
| --- | --- | --- |
| *YWHAZ* | CCTGCATGAAGTCTGTAACTGAG | GACCTACGGGCTCCTACAACA |
| *TFCP2* | CTTCCTATGAGACAACCAT | ATGGTTGTCTCATAGGAAG |
| *SULF1* | GAAGGAGAAGAGACGGCAGA | CAGAAAGATCCCAGGTTCCA |
| *HS6ST2* | CCGTCCAGGAACTTCCACTA | GACCAGTCATCGCCAGTGTA |
| *GPC4* | CAGCAGTGCCCTTCAGAGTT | ACTGTCGGCTTTCTCATTGG |
