# Supplementary material for: TFCP2 is a transcriptional regulator of heparan sulfate assembly and melanoma cell growth
Source: J Biol Chem. 2023 Apr 13;299(6):104713. doi: 10.1016/j.jbc.2023.104713 (PMC10200990; doi:10.1016/j.jbc.2023.104713)
Supplement: Supporting Figure S1 — A375 TFCP2 knockout clones.A, CRISPR sgRNA targeting of human TFCP2 in A375 cells. Frameshift biallelic mutations in exon 1 were confirmed in clone 21 and clone 9 by Sanger sequencing (introns shown as gray text). Brightfield images of (B) A375 wildtype and (C) TFCP2 clone 21 show minimal differences in cell morphology. Scale Bar = 360 μm. [file mmc3.pdf]

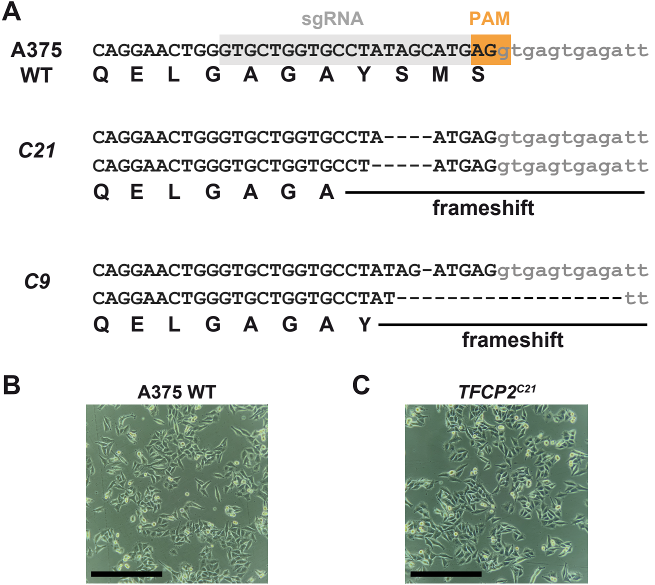
A
sgRNA
PAM
A375 WT
CAGGAACTGGGTGCTGGTGCCTATAGCATGAGgtgagtgagatt
Q E L G A G A Y S M S
C21
CAGGAACTGGGTGCTGGTGCCTA----ATGAGgtgagtgagatt
CAGGAACTGGGTGCTGGTGCCT-----ATGAGgtgagtgagatt
Q E L G A G A
frameshift
C9
CAGGAACTGGGTGCTGGTGCCTATAG-ATGAGgtgagtgagatt
CAGGAACTGGGTGCTGGTGCCTAT------------------tt
Q E L G A G A Y
frameshift
B
A375 WT
C
TFCP2[C21]
