# Supplementary figures and images for: TFCP2 is a transcriptional regulator of heparan sulfate assembly and melanoma cell growth
Source: J Biol Chem. 2023 Apr 13;299(6):104713. doi: 10.1016/j.jbc.2023.104713 (PMC10200990; doi:10.1016/j.jbc.2023.104713)

**A**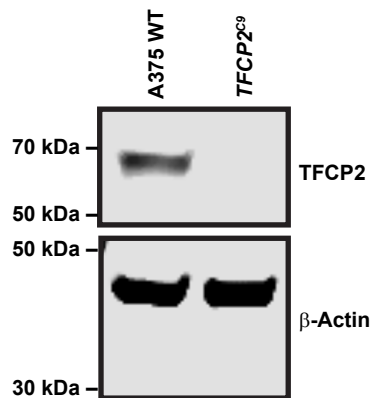**B**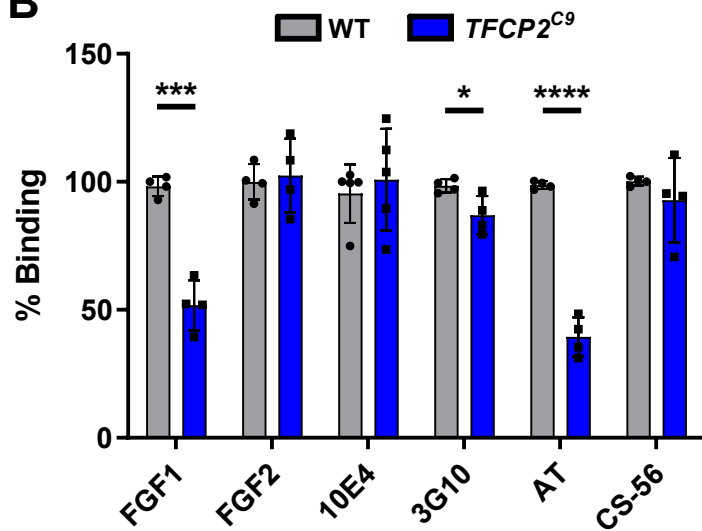**C**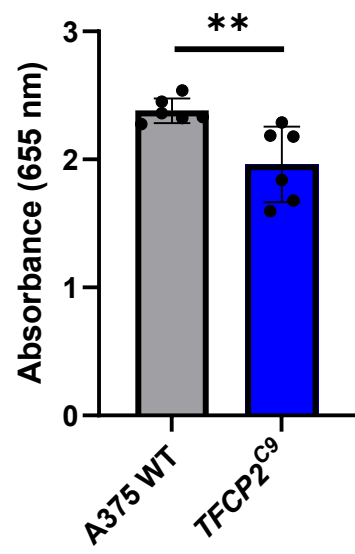**D**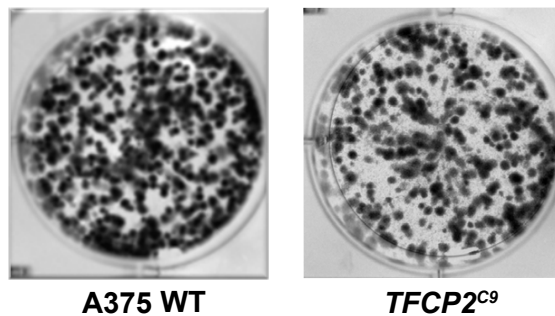

Supplement: Supporting Figure S2 — A375 TFCP2 knockout clone C9 characterization.A, Western blot for TFCP2 knockout clone 9 in A375 cells. B, Ligand binding in A375 TFCP2 knockout clone C9 is comparable to clone C21 (t-tests; n ≥ 4, ∗p < 0.05, ∗∗∗p < 0.001, ∗∗∗∗p < 0.0001). C and D, TFCP2 knockout clone C9 exhibits reduced growth in clonogenic assays, as quantified by methylene blue staining and absorption readings at 655 nm (t-test, n = 3, ∗∗p < 0.01). Note: the A375 wildtype representative image shown is reused from Figure 5C, as clonogenic assays for both TFCP2 knockout clones were performed in parallel and compared to the same wildtype samples for each independent biological replicate experiment. [file mmc4.pdf]

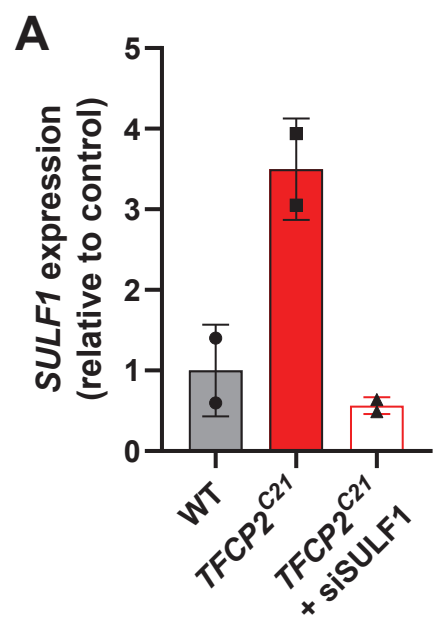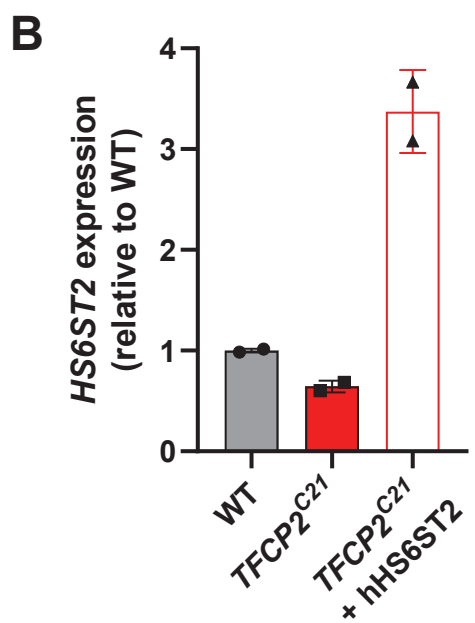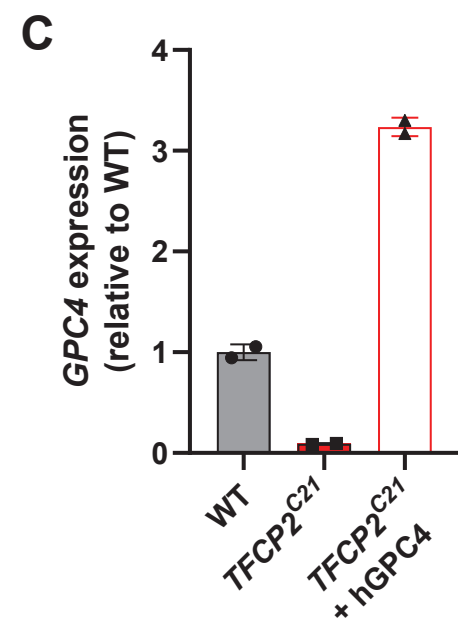

Supplement: Supporting Figure S3 — Quantitative PCR data for rescue experiments.A, SULF1 mRNA expression in A375 TFCP2C21 cells is restored to wildtype levels upon transfection of a silencing RNA targeting SULF1 (siSULF1) (n = 2 independent experiments). B, Fold change in HS6ST2 mRNA expression in A375 TFCP2C21 cells and in A375 TFCP2C21 cells expressing HS6ST2 cDNA from a lentiviral construct (hHS6ST2) (n = 2 independent experiments). C, Fold change in GPC4 mRNA expression in A375 TFCP2C21 cells, and in A375 TFCP2C21 cells expressing GPC4 cDNA from an expression plasmid (hGPC4) (n = 2 independent experiments). [file mmc5.pdf]

**A**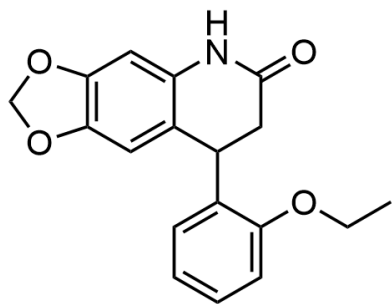**B**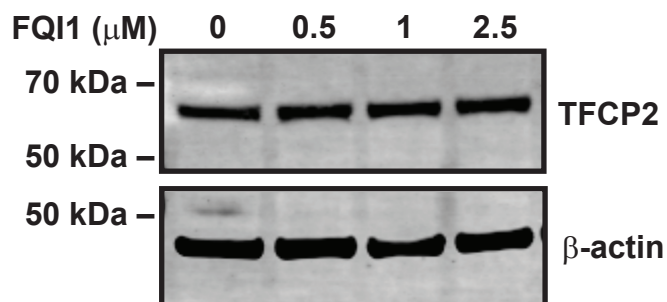**C**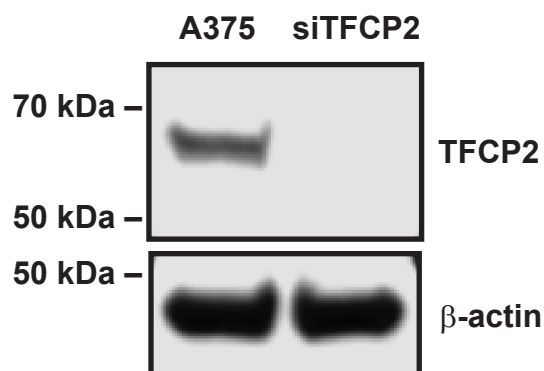**D**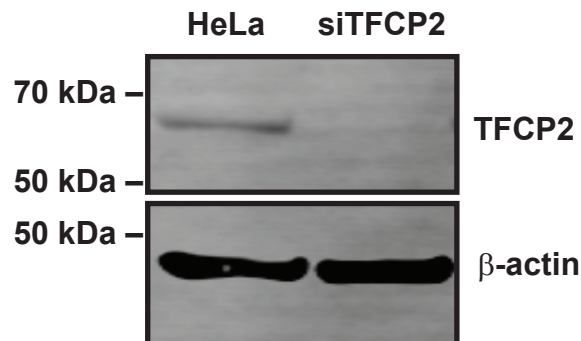**E**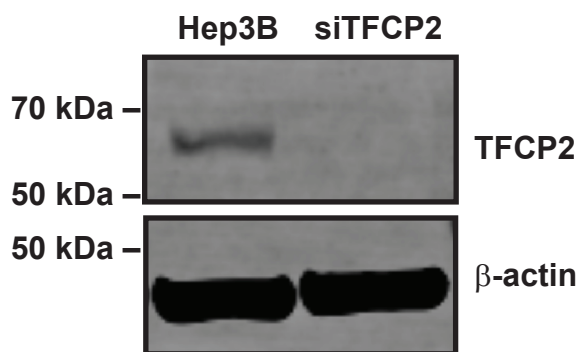**F**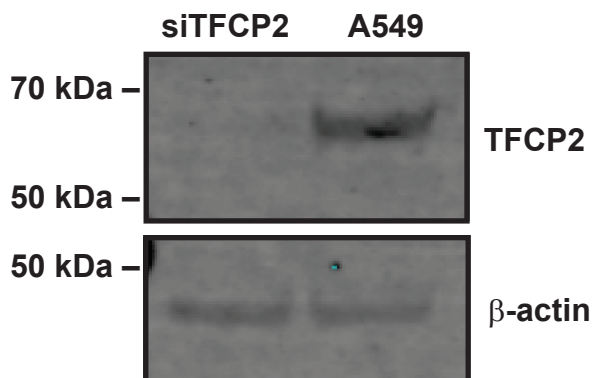**G**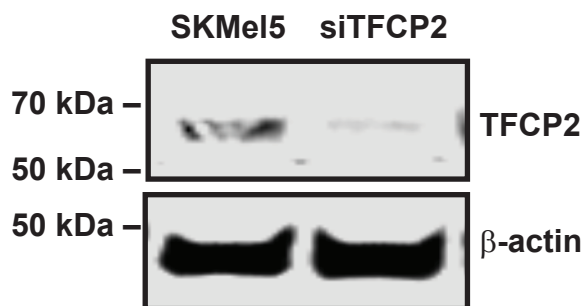**H**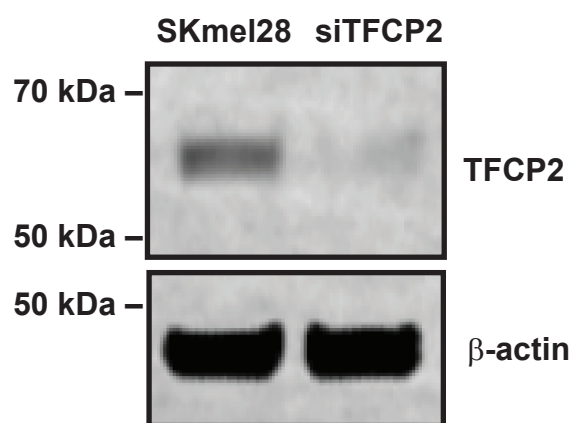**I**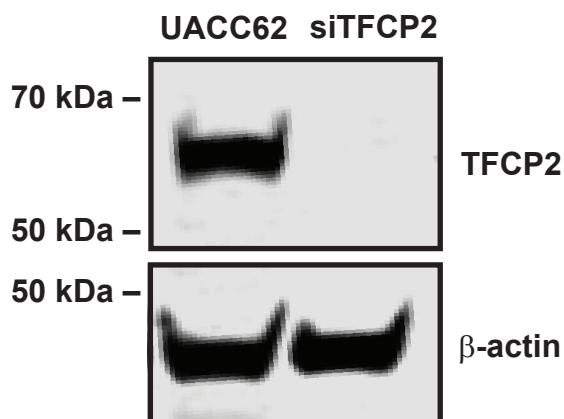**J**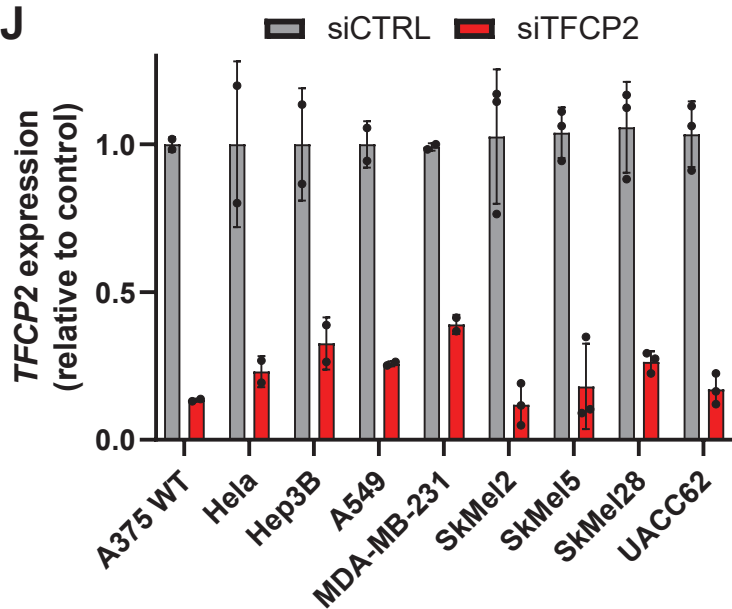

Supplement: Supporting Figure S4 — Targeting TFCP2 in other human cell lines.A, Chemical structure for the TFCP2 inhibitor, factor quinolinone inhibitor 1 (FQI1). B, Western blot reveals no change in TFCP2 protein levels in A375 cells treated with various concentrations of FQI1 for 24 h. Western blot images show silencing of TFCP2 expression in (C) A375, (D) HeLa, (E) Hep3B, (F) A549, (G) SKMel5, (H) SKMel28, (I) UACC62 cell lines. Images are representative of two independent experiments. J, Quantitative PCR measuring TFCP2 expression upon transfection of siRNA targeting TFCP2 in human cell lines (n ≥ 2 independent experiments). [file mmc6.pdf]

**A**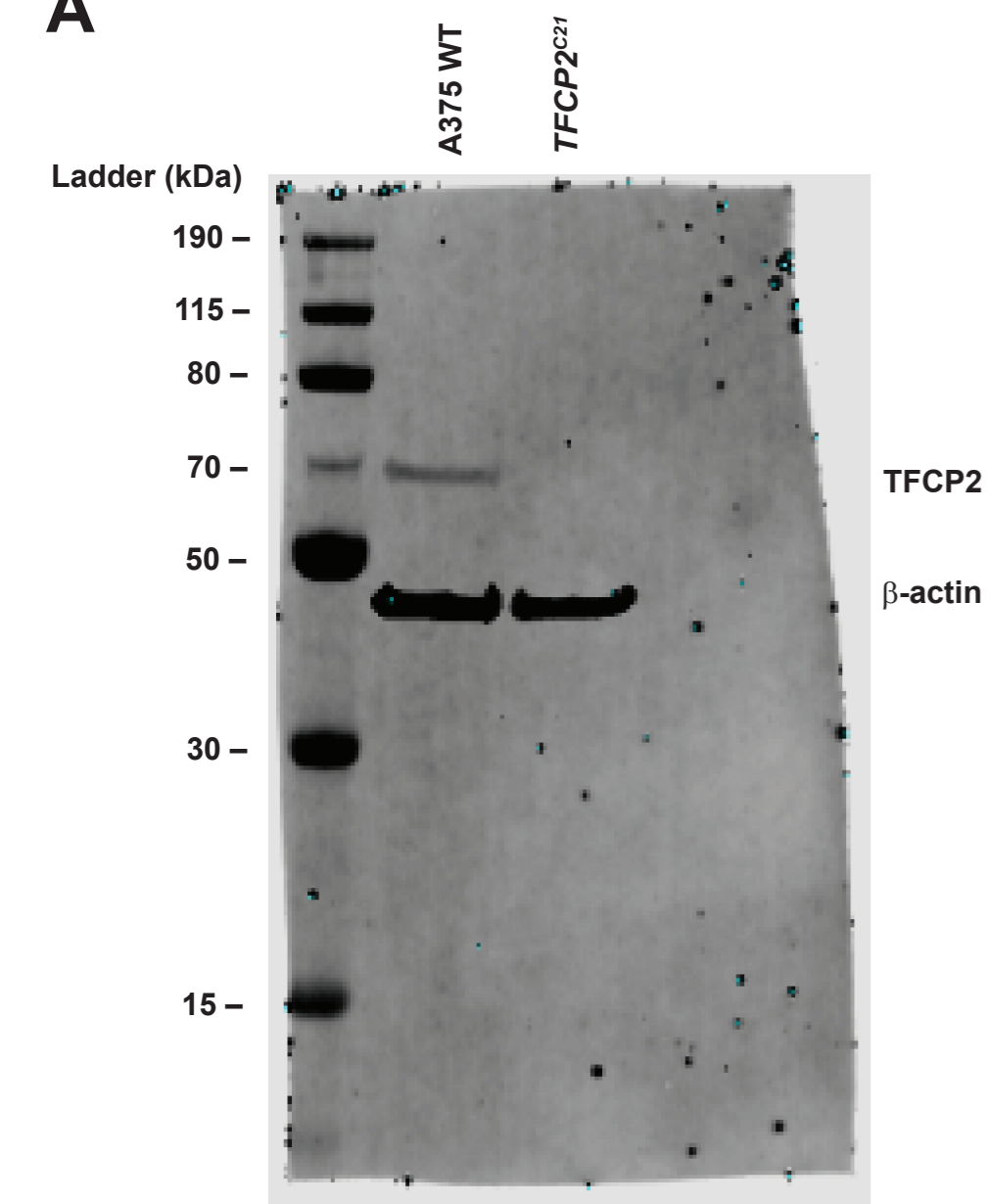**B**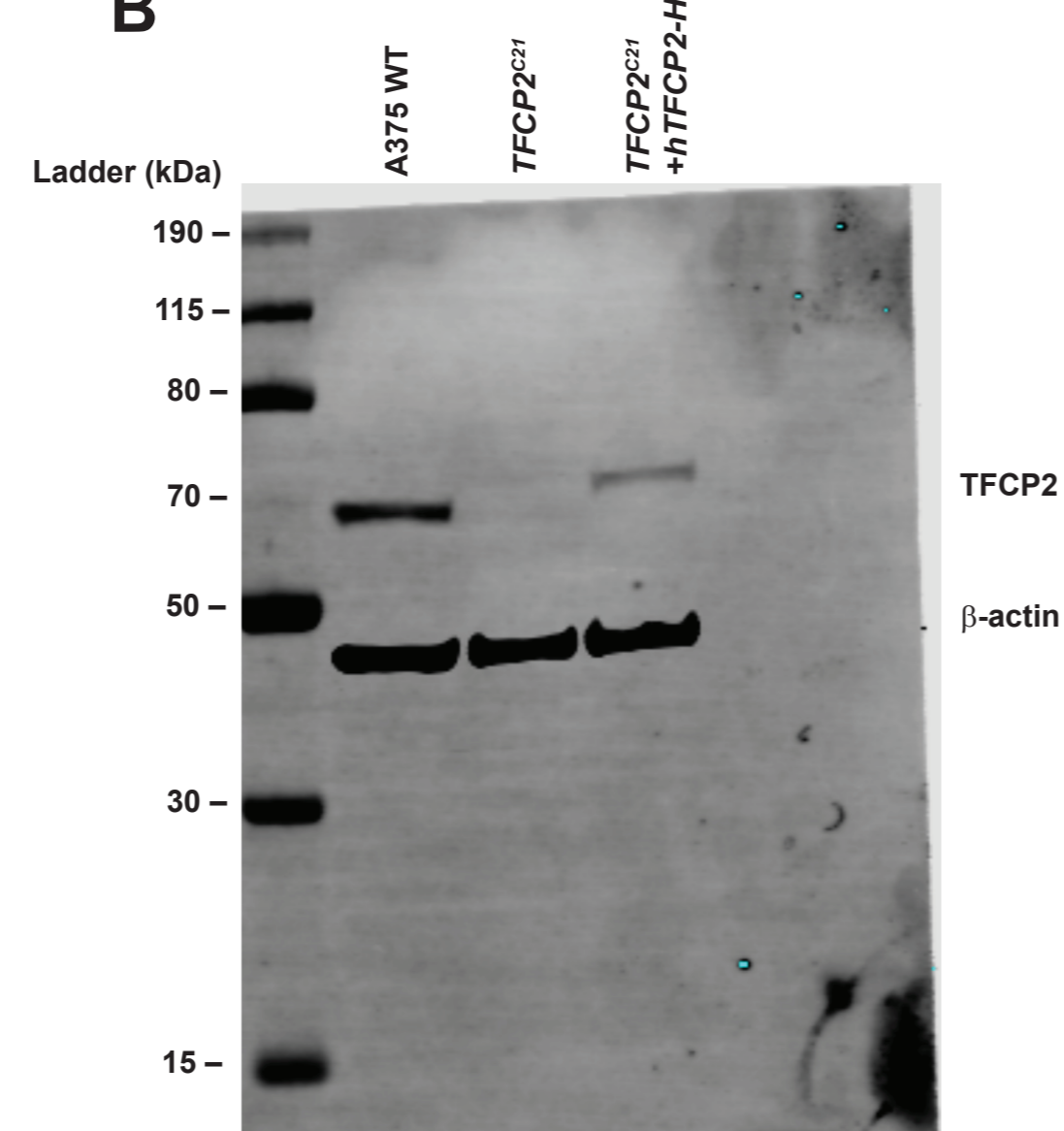**C**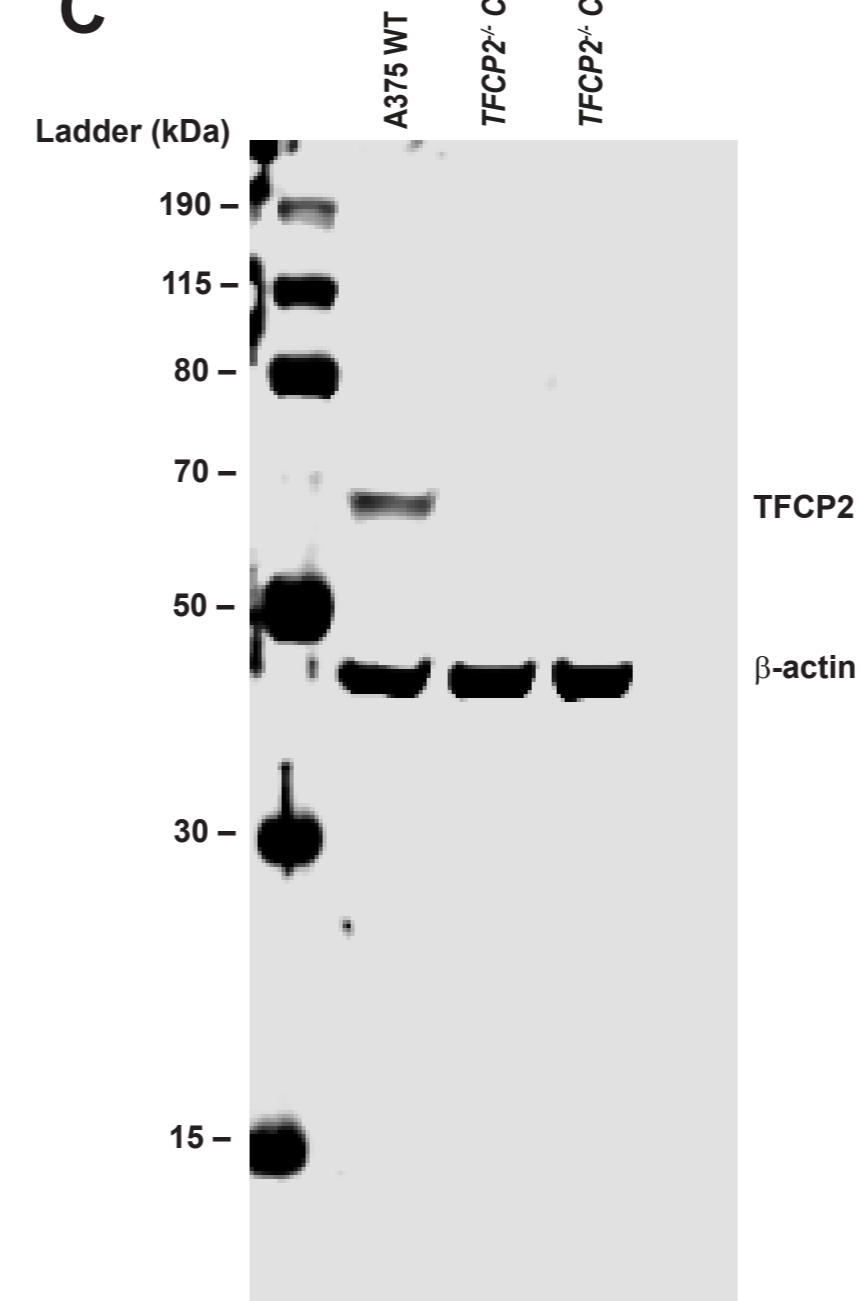**D**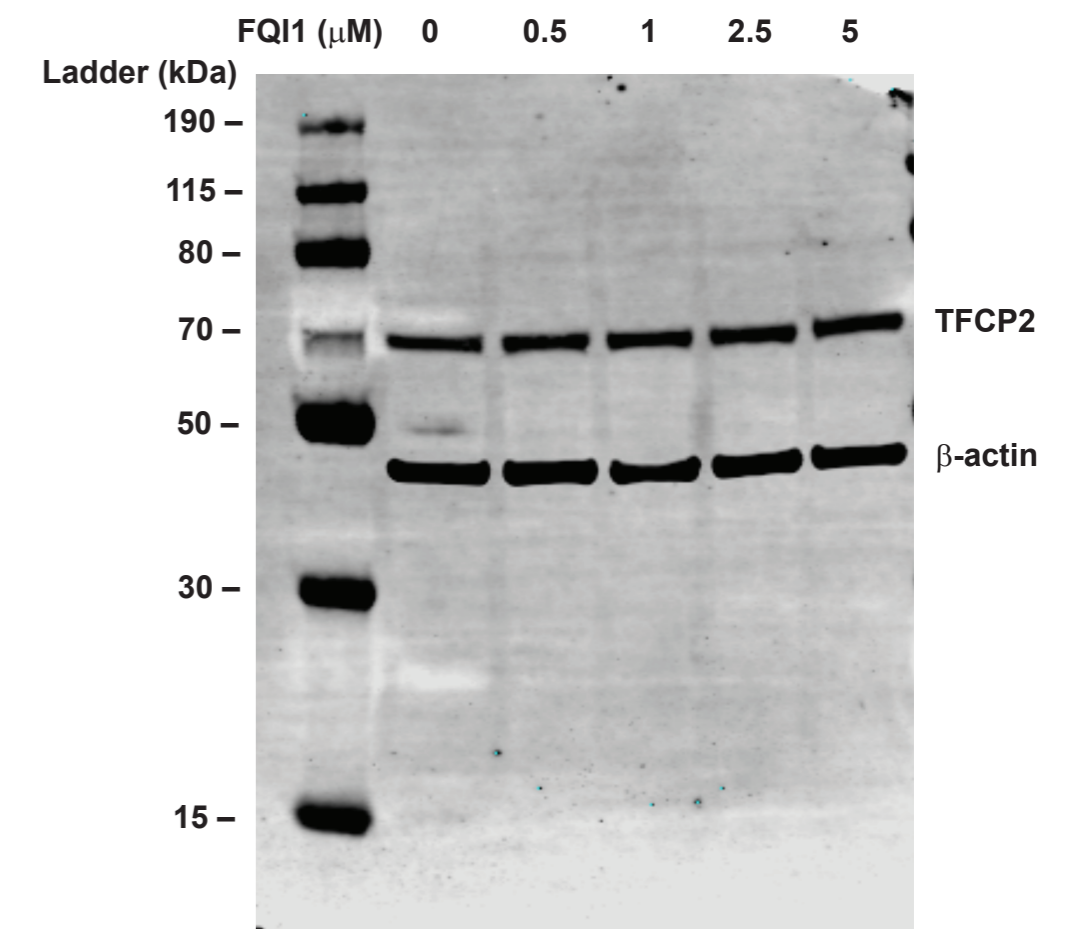**E**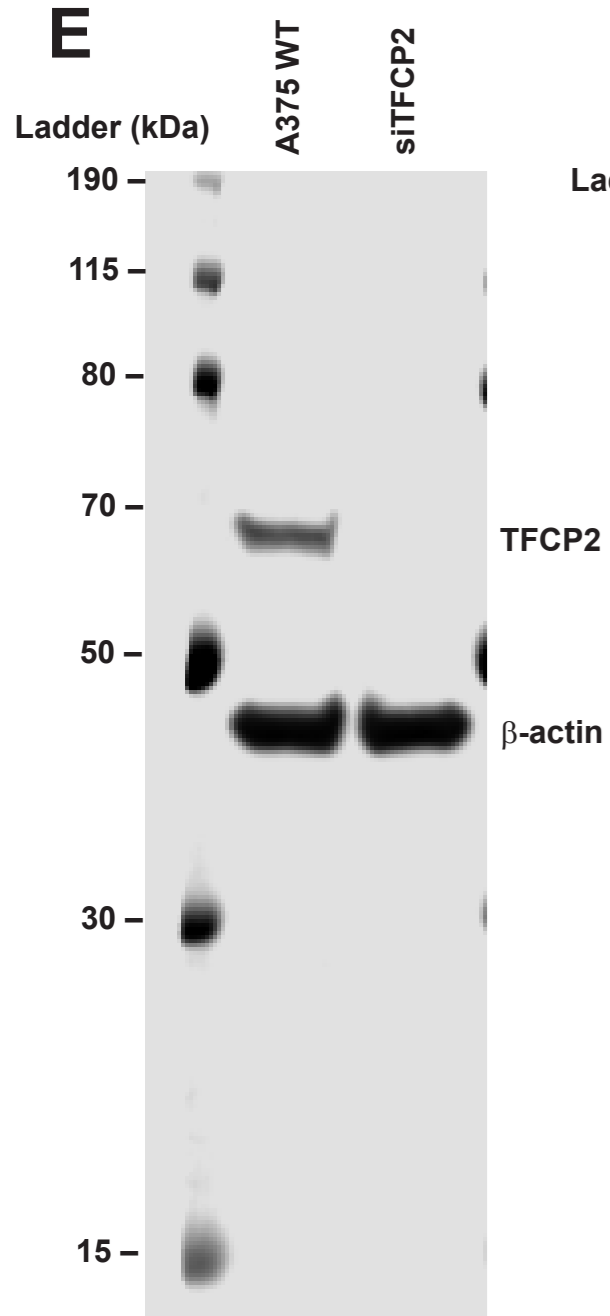**F**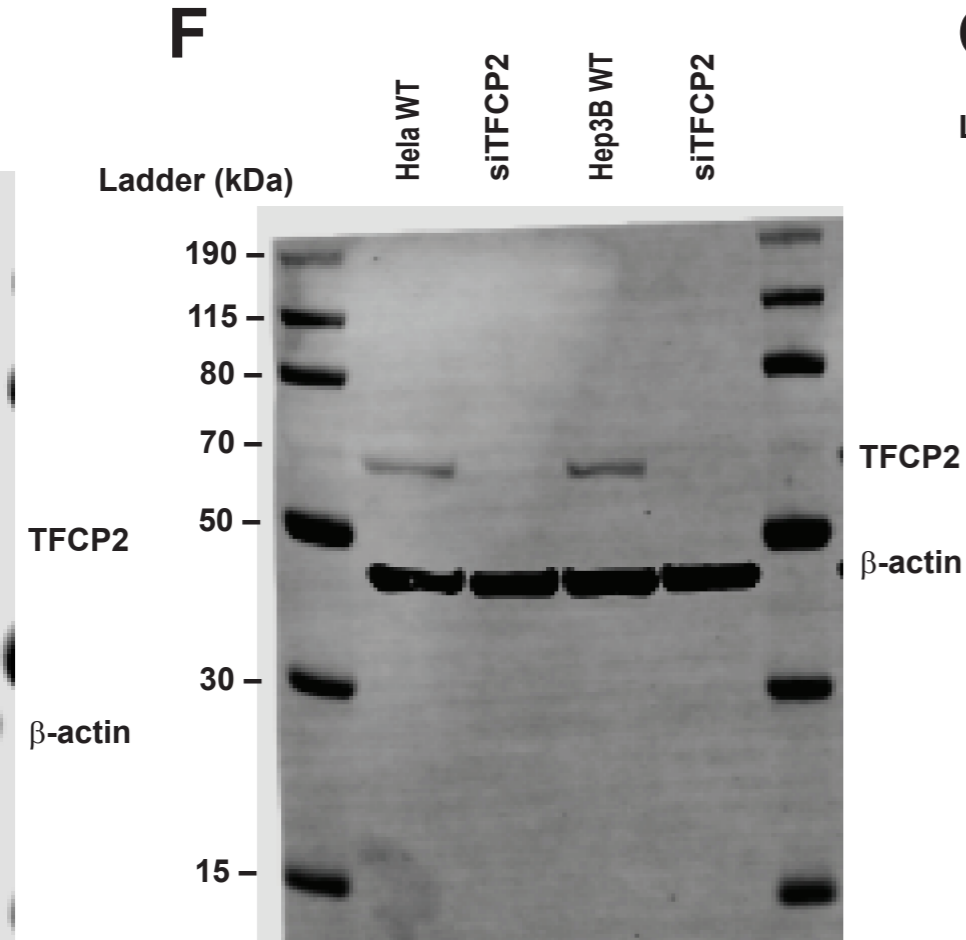**G**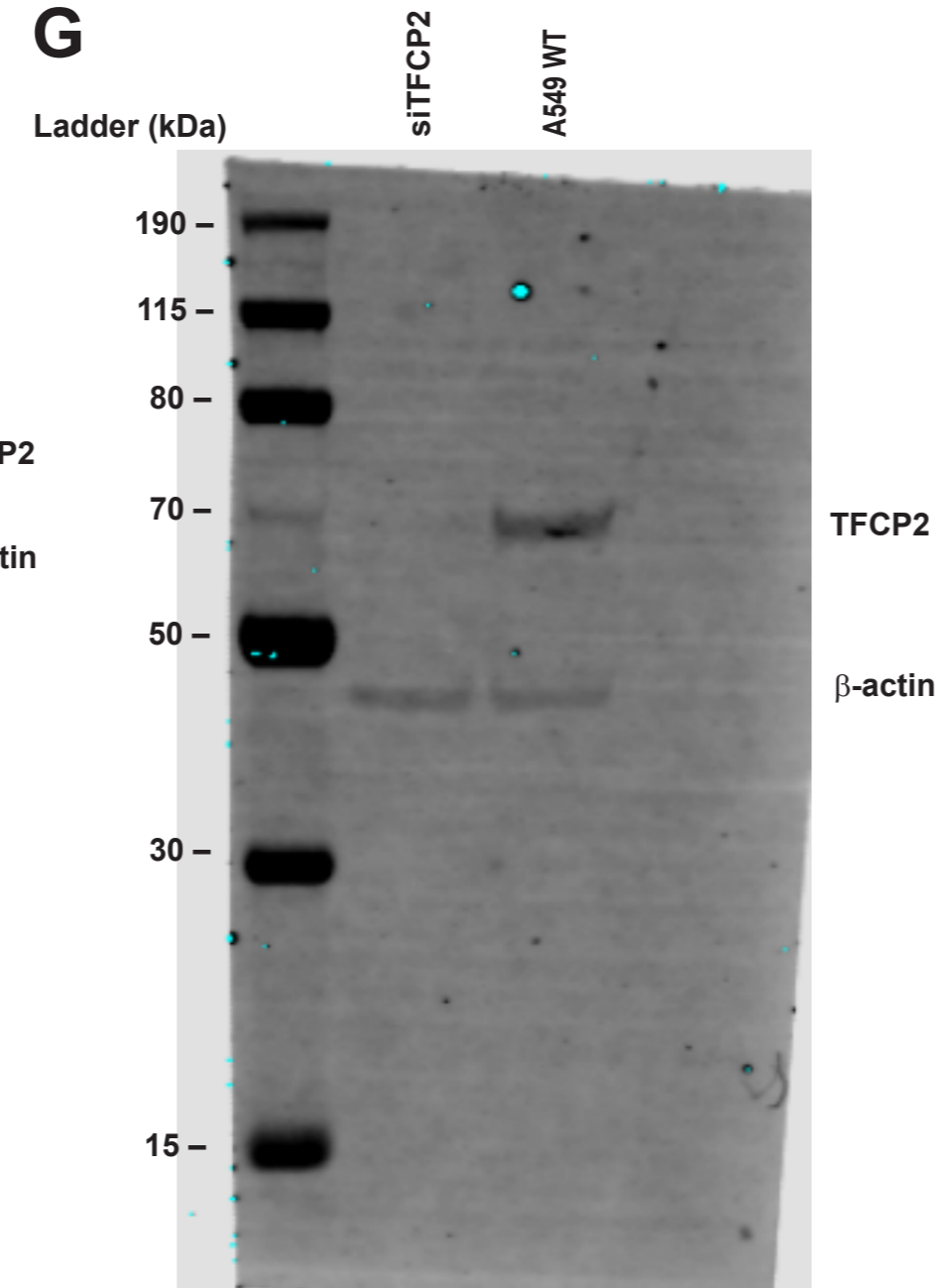**H**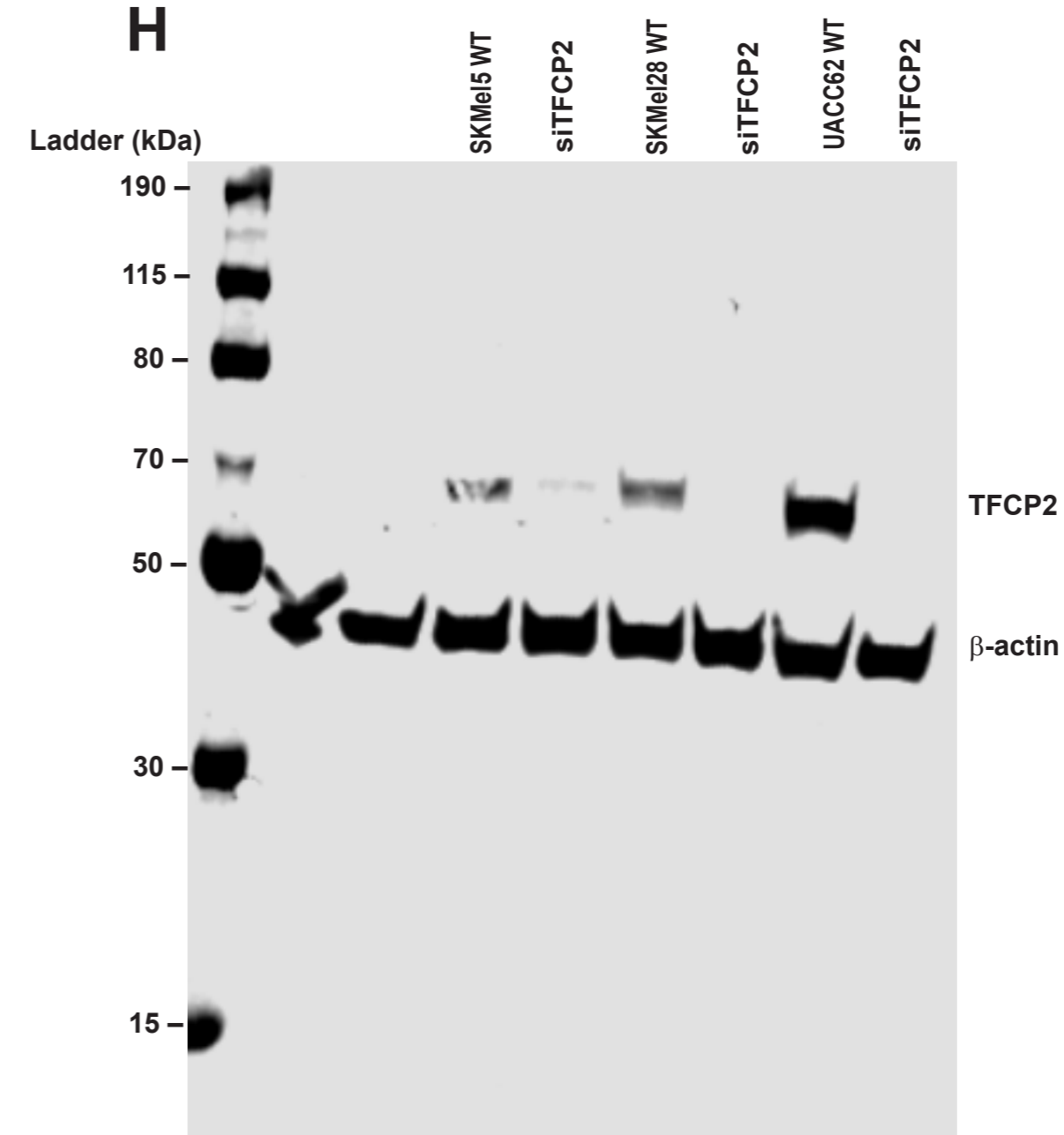**I**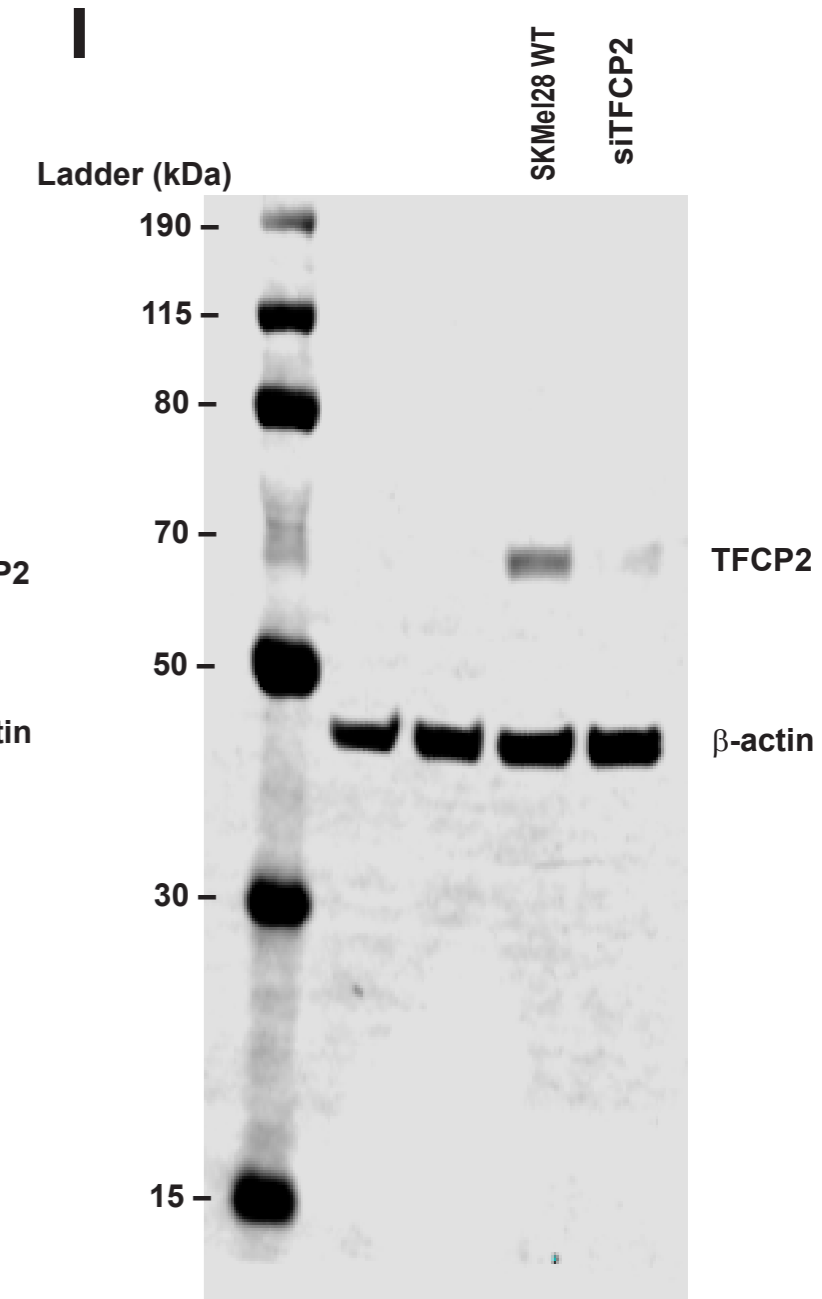

Supplement: Source Data Figure — Full western blot source images for (A) Figure 1B, (B) Figure 2B, (C) Fig S2A, (D) Fig S4B, (E–I) Fig S4, A–G. [file mmc7.pdf]
